# Yellow cerebrospinal fluid in an extremely preterm infant: a case report

**DOI:** 10.3389/fncel.2026.1792995

**Published:** 2026-04-10

**Authors:** Xiyang Chen, Haiting Li, Jie Li, Linlin Chen, Xixi Liu, Dengpan Xie, Yunqin Chen, Junhui Yuan, Enfu Tao

**Affiliations:** Department of Neonatology and NICU, Wenling Maternal and Child Health Care Hospital, Wenling, Zhejiang, China

**Keywords:** bilirubin, bilirubin encephalopathy, cerebrospinal fluid, extremely preterm infant, neurodevelopmental outcome, phototherapy

## Abstract

Although phototherapy has reduced the incidence of kernicterus in term infants, it remains a significant threat to extremely preterm infants due to their immature blood–brain barrier and frequent comorbidities such as sepsis. Current clinical practice relies on serum total bilirubin levels, which may not accurately reflect cerebral bilirubin exposure in this vulnerable population. This case report describes an extremely preterm infant (gestational age 28 1/7 weeks, birth weight 950 g) who developed visibly yellow cerebrospinal fluid (CSF) on the first day of life. Despite only minimal serum hyperbilirubinemia (4.4 mg/dL; ≈ 75.2 μmol/L), CSF bilirubin was markedly elevated at 10 mg/dL (≈ 171 μmol/L). The infant presented with respiratory distress syndrome and sepsis. Immediate intensive phototherapy was initiated, leading to the normalization of CSF bilirubin (3.6 mg/dL ≈ 61.5 μmol/L) within 4 days. Brain magnetic resonance imaging (MRI) and automated auditory brainstem response (AABR) performed at the corrected age of 4 months were both unremarkable. At the corrected age of 12 months, neurodevelopmental assessment using the Bayley Scales of Infant Development-III (BSID-III) showed scores within the normal range. This case illustrates that CSF bilirubin can serve as a sensitive early biomarker for identifying preterm infants at imminent risk for bilirubin neurotoxicity, particularly when serum bilirubin levels are misleadingly low. Targeted measurement of CSF bilirubin in selected high-risk infants, when a lumbar puncture is otherwise indicated, could enable more timely intervention and contribute to improved neurodevelopmental outcomes.

## Introduction

Bilirubin-induced neurological injury, also known as bilirubin encephalopathy, results from the neurotoxicity of unconjugated bilirubin after it crosses the immature blood–brain barrier (Qian et al., 2022). Within the central nervous system, bilirubin can disrupt cellular energy metabolism, induce oxidative stress, and trigger neuronal apoptosis, particularly in vulnerable regions such as the basal ganglia, brainstem nuclei, and auditory pathways (Hansen et al., 2020). Clinically, the spectrum ranges from acute manifestations—including lethargy, hypertonia, and opisthotonos—to chronic sequelae such as sensorineural hearing loss (often detected by abnormal automated auditory brainstem response, AABR), movement disorders including dystonia or athetosis, and gaze abnormalities. Magnetic resonance imaging (MRI) may demonstrate characteristic signal abnormalities, typically T1 or T2 hyperintensity in the globus pallidus and subthalamic nuclei (Qian et al., 2022; Wisnowski et al., 2014).

Despite advances in phototherapy, kernicterus remains a significant threat to extremely preterm infants, particularly in settings where serum bilirubin may underestimate cerebral bilirubin exposure (Qattea et al., 2021). Globally, and especially in low- and middle-income countries, kernicterus still accounts for a substantial proportion of preventable neonatal mortality and morbidity (Wennberg et al., 2023; Diala et al., 2018). Bilirubin-induced neurologic dysfunction (BIND), encompassing the spectrum from kernicterus to auditory pathway injury, continues to affect newborns with hyperbilirubinemia (Ahlfors et al., 2009).

Extremely preterm infants are at heightened risk due to inherently immature blood–brain barrier (BBB) permeability and a high frequency of comorbidities such as sepsis that further disrupt barrier integrity (Nawaz et al., 2021). Traditional management relies on serum bilirubin thresholds, which may poorly reflect actual brain bilirubin deposition in preterm infants with variable and dynamic BBB function (Hulzebos et al., 2014). Cerebrospinal fluid bilirubin is considered a more direct indicator of central nervous system (CNS) bilirubin load, as it directly reflects the level of free bilirubin that has crossed the BBB and entered the central nervous system (Shukla et al., 1988; Kulkarni et al., 1989). However, data in extremely preterm infants are scarce, and its clinical application remains controversial due to the invasiveness of the required lumbar puncture.

This case report describes an extremely preterm infant who developed profoundly yellow CSF due to markedly elevated bilirubin, which was successfully managed with intensive phototherapy. Despite extreme CSF bilirubin elevation, the infant achieved favorable neurodevelopmental outcomes. This case challenges conventional serum-based intervention paradigms and underscores the potential critical role of CSF bilirubin assessment in guiding treatment decisions for high-risk preterm infants. We review the underlying pathophysiology, discuss the integrated therapeutic strategy, and explore the long-term implications based on current literature, aiming to contribute to the refinement of neuroprotective protocols for this vulnerable population.

## Case presentation

A female infant was born via cesarean section at 28 1/7 weeks of gestation to a 24-year-old primigravida with preterm premature rupture of membranes (PPROM) for >20 days. Upon admission, the mother received empirical oral amoxicillin for 4 days. Subsequent vaginal cultures grew multidrug-resistant, extended-spectrum beta-lactamase (ESBL)–producing *Escherichia coli* (*E. coli*). Based on antimicrobial susceptibility testing, antibiotic therapy was escalated to intravenous cefoperazone–sulbactam. A cesarean section was performed 18 h after initiation of cefoperazone–sulbactam therapy. A complete course of antenatal steroids was administered. Birth weight was 950 g (appropriate for gestational age). Apgar scores were 6 at 1 min and 7 at 5 min. The infant was transferred to the neonatal intensive care unit (NICU) due to extreme prematurity and respiratory distress.

Upon admission, the infant exhibited tachypnea (respiratory rate 66 breaths/min), grunting, intercostal retractions, poor activity, and weak primitive reflexes. Chest X-ray confirmed respiratory distress syndrome (RDS) with grade II changes. Initial laboratory tests revealed normoglycemia (2.9 mmol/L) and an elevated procalcitonin level (0.75 ng/mL). The complete blood count (CBC) showed a leukocyte count of 8.0 × 10^9^/L with marked neutropenia (neutrophils 19.7%) and relative lymphocytosis (lymphocytes 68.1%). High-sensitivity c-reactive protein (hs-CRP) was < 0.5 mg/L. These findings, in conjunction with the maternal history of ESBL *E. coli* colonization and preterm premature rupture of membranes, raised concern for early-onset sepsis (EOS). She received invasive mechanical ventilation, surfactant therapy (poractant alfa, 200 mg/kg), and empirical antibiotics (penicillin and cefotaxime). All initial blood test results are listed in Table 1.

**Table 1:** Key laboratory findings on admission.

Investigation	Results	Reference range
Complete blood count		
White blood cell, ×10^9^/L	8.0	15 ~ 20
Red blood cell, × 10^9^/L	4.56	6 ~ 7
Hematocrit, %	56.6	35 ~ 65
Hemoglobin (Hb), g/l	176	170 ~ 200
Platelet count (PLT), ×10^9^/L	233	100 ~ 300
Neutrophil percentage, %	19.7	20 ~ 40
Lymphocytes percentage, %	68.1	50 ~ 75
C-reactive protein, mg/L	< 0.5	0 ~ 5.0
Blood gas		
pH	7.31	7.35 ~ 7.45
PaCO₂, mmHg	48.6	35 ~ 45
PaO₂	74.7	80 ~ 100
Actual bicarbonate, mmol/L	21.5	18.0 ~ 26.0
Base excess, mmol/L	−3.12	−3.0 ~ +3.0
Lactic acid, mmol/L	3.3	0.5 ~ 2.2
Sodium ion, mmol/L	135.3	135 ~ 145
Potassium ion, mmol/L	4.04	3.5 ~ 5.5
Calcium ion, mmol/L	1.26	1.05 ~ 1.35
Chloride ion, mmol/L	104.5	98 ~ 113
Blood glucose, mmol/L	4.0	3.9 ~ 6.1
Blood biochemistry		
Sodium ion, mmol/L	139	135 ~ 145
Potassium ion, mmol/L	4.32	3.5 ~ 5.5
Total calcium, mmol/L	2.24	2.25 ~ 2.75
Chloride ion, mmol/L	108	99 ~ 110
Inorganic phosphate, mmol/L	2.31	1.45 ~ 2.1
Magnesium, mmol/L	0.94	0.75 ~ 1.02
Albumin, g/l	29	28 ~ 44
Hepatorenal function	Normal	Normal

pH, potential of hydrogen; PaCO₂, partial pressure of carbon dioxide; PaO₂, partial pressure of oxygen.

**Figure 1:**
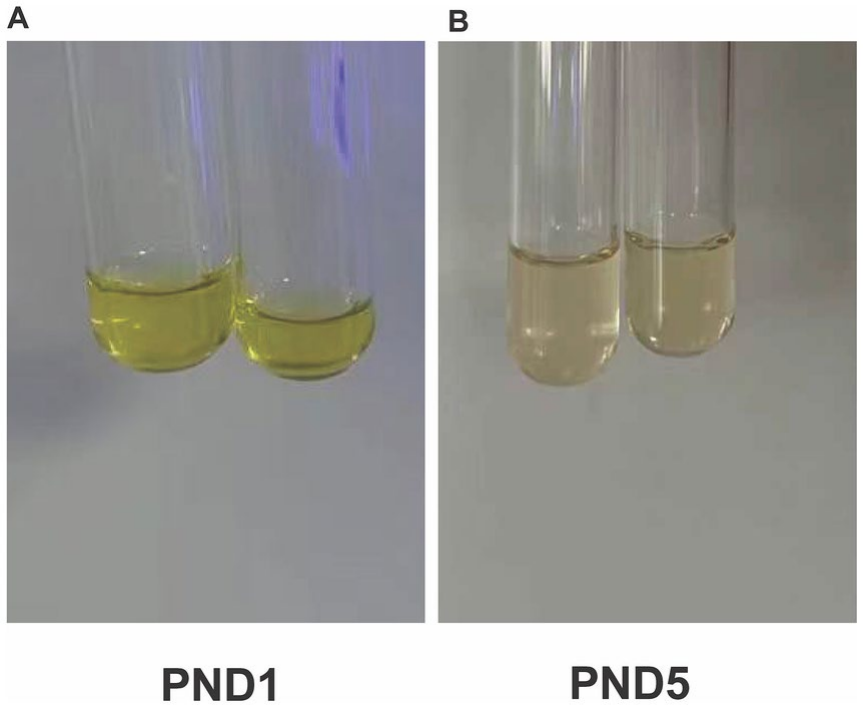
Rare macroscopic xanthochromia of cerebrospinal fluid in an extremely preterm neonate. **(A)** Marked yellow discoloration of cerebrospinal fluid observed on PND1. **(B)** Clear and colorless cerebrospinal fluid observed on postnatal day 5. PND, postnatal day.

**Figure 2:**
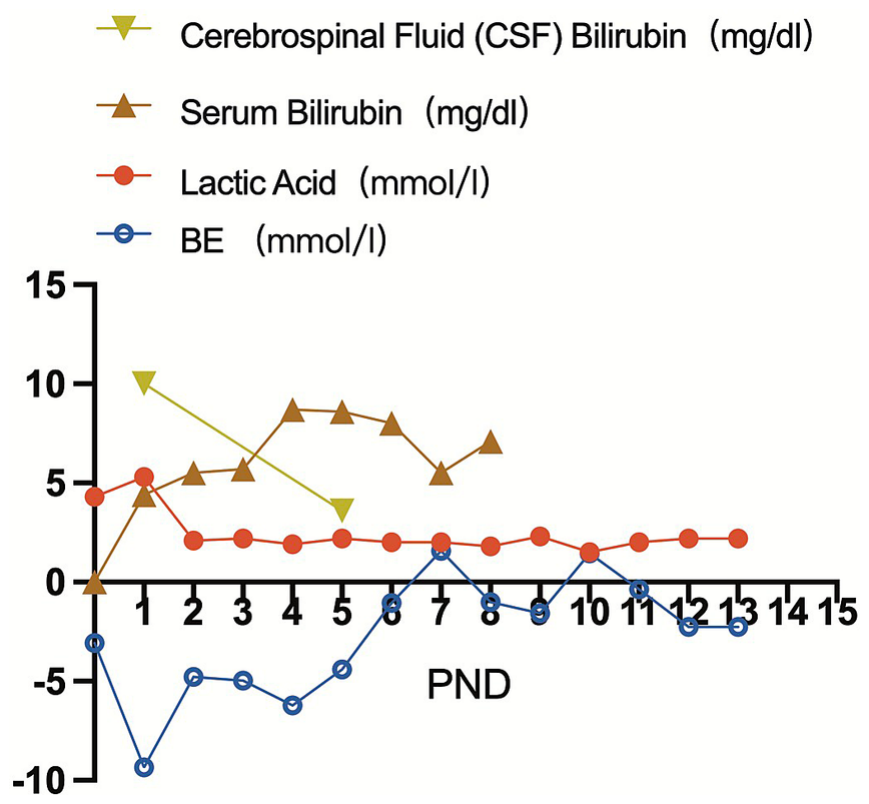
Temporal changes in cerebrospinal fluid bilirubin, serum bilirubin, lactate, and base excess after birth. Serial postnatal trends of cerebrospinal fluid (CSF) bilirubin, serum bilirubin, lactate, and base excess (BE) are shown. A striking divergence is evident: CSF bilirubin peaked early (10 mg/dL on PND 1) and declined rapidly following intervention, while serum bilirubin exhibited a delayed, more gradual rise. Lactate and BE values mirrored the clinical course of septic shock and subsequent recovery. PND, postnatal day.

Bilirubin was monitored daily via transcutaneous bilirubinometry (TCB). On postnatal day (PND) 1, TCB was 10 mg/dL. Given the extreme prematurity and concomitant sepsis, and in accordance with the principle that preterm infants require lower intervention thresholds due to heightened neurotoxicity risk, intensive phototherapy was initiated immediately (Bhutani and Wong, 2013; Kemper et al., 2022). Concurrently, the infant developed signs of clinical sepsis, including fever (38.3 °C) and hypotension. Given the clinical instability and concern for purulent meningitis, a diagnostic lumbar puncture was performed on PND 1 under strict aseptic conditions. Using a standard lumbar puncture kit, the needle was inserted at the L3–L4 intervertebral space. Strikingly, the CSF appeared visibly yellow yet remained clear (Figure 1A). The CSF was immediately divided into three tubes. The first tube was sent for microbiological examination, including culture and smear. The second tube was used for routine and biochemical analyses, including cell count, protein, and glucose measurements. The third tube was reserved for bilirubin determination. For bilirubin measurement, the CSF sample was analyzed within 1 h after collection under light-protected conditions using an automated clinical chemistry analyzer (Siemens Healthineers). Routine analysis showed no signs of intracranial infection, with a white blood cell count of 0/μL and a protein level of 52 mg/dL. Due to the pronounced xanthochromia, CSF bilirubin level was measured and found to be markedly elevated at 10 mg/dL (≈171 μmol/L), whereas the concurrent serum total bilirubin was only 4.4 mg/dL (≈75.2 μmol/L). An intensified phototherapy regimen was immediately implemented using enhanced four-sided lights. Given the concurrent septic shock, hemodynamic instability, and extreme prematurity of the infant, exchange transfusion was deferred. Albumin infusion was administered to increase colloid oncotic pressure and bind free bilirubin, while intensive phototherapy and comprehensive supportive care were continued. Following this management, the metabolic acidosis resolved, as evidenced by a decrease in lactate from 5.3 mmol/L to 1.5 mmol/L (reference range: 0.5–2.2 mmol/L) and a normalization of base excess (BE) from −9.3 mmol/L to −1.5 mmol/L (reference range: −3 – +3 mmol/L) by PND 5 (Figure 2). Serum bilirubin levels began to decline within 24 h. A repeat lumbar puncture on PND 5 showed that the CSF bilirubin level had decreased to 3.6 mg/dL, with the CSF color returning to clear (Figure 1B). Serum bilirubin at this time was 8.6 mg/dL. Intermittent phototherapy was continued until day 10, by which time TCB had stabilized below 5 mg/dL. The temporal changes in cerebrospinal fluid bilirubin, serum bilirubin, lactate, and base excess after birth are shown in Figure 2.

The infant’s hospital course was complicated by multiple preterm comorbidities. She required invasive mechanical ventilation from days 1 to 8, followed by non-invasive support. A recurrence of respiratory distress necessitated brief reintubation from days 17 to 20, after which she was successfully weaned to non-invasive support and subsequently to continuous positive airway pressure (CPAP), eventually tolerating room air at discharge. For infection management, antibiotics were escalated to piperacillin-tazobactam and vancomycin upon development of septic shock, with aggressive fluid and inotropic support. Although the infant’s blood cultures were negative, the presence of prolonged rupture of membranes in the mother, the isolation of multidrug-resistant *Escherichia coli* from vaginal secretion cultures, and the development of clinical features consistent with septic shock after birth collectively support the diagnosis of clinical sepsis [World Health Organization (WHO), 2024]. Antibiotic therapy was continued for a total of 14 days, with all CSF cultures remaining sterile. Echocardiography confirmed a hemodynamically significant patent ductus arteriosus (PDA). Treatment with ibuprofen was initiated on day of life 6, together with fluid restriction to 140 mL/kg/day to facilitate ductal closure. The PDA closed successfully by day 20. Other complications included anemia requiring red blood cell transfusion, mild retinopathy of prematurity (which resolved spontaneously), and feeding intolerance managed with gradual advancement of enteral nutrition.

**Figure 3:**
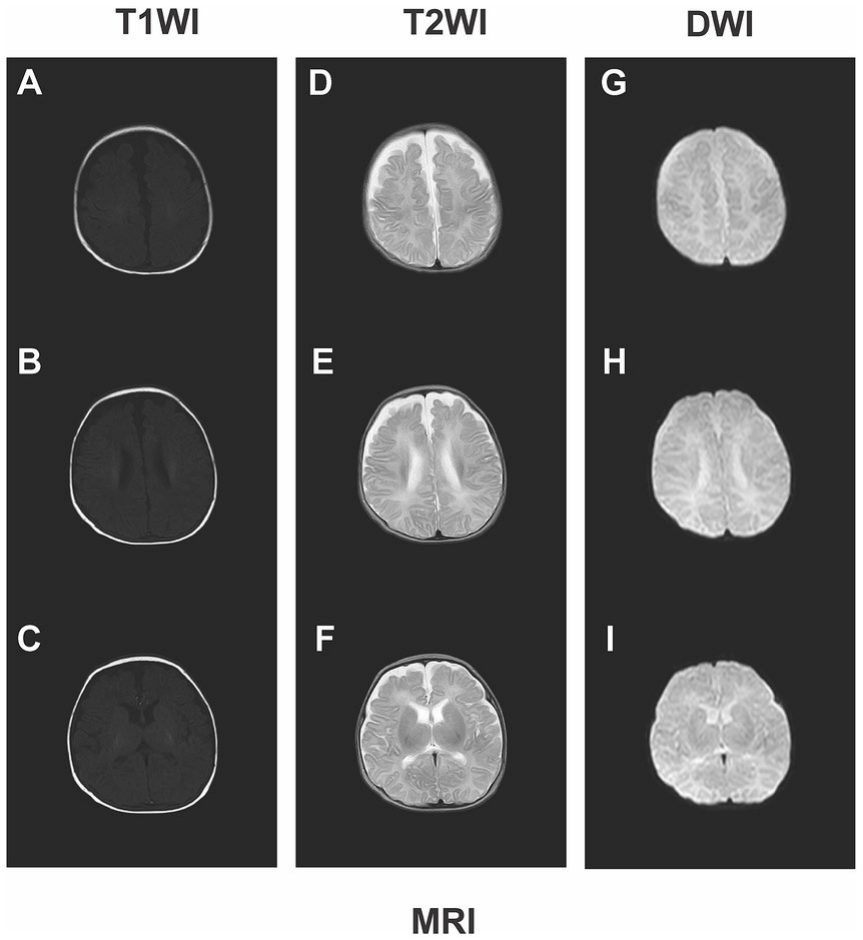
Brain magnetic resonance imaging at four months of age shows normal structure. To assess long-term prognosis, brain MRI was performed at four months of age. Axial T1-weighted **(A–C)**, T2-weighted **(D–F)**, and diffusion-weighted **(G–I)** images depict the levels of the centrum semiovale **(A,D,G)**, periventricular region **(B,E,H)**, and basal ganglia **(C,F,I)**, respectively. No structural or signal abnormalities indicative of prior bilirubin-induced neurological injury were identified on any sequence.

**Figure 4:**
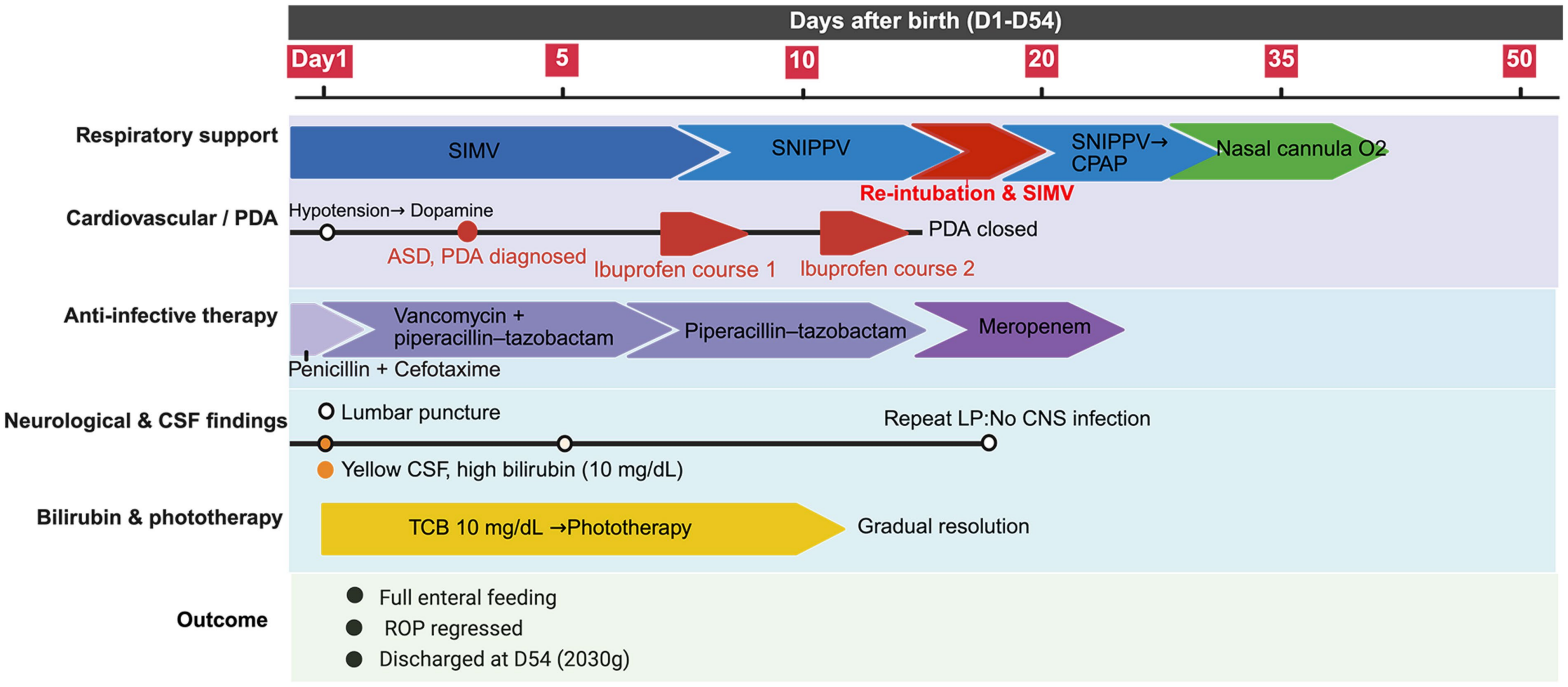
Timeline of clinical course and management. The figure illustrates the chronological progression of respiratory support, cardiovascular management, anti-infective therapy, neurological evaluation, and bilirubin-related interventions from birth to discharge. Key diagnostic and therapeutic milestones are highlighted along the timeline. Abbreviations: SIMV, synchronized intermittent mandatory ventilation; SNIPPV, synchronized nasal intermittent positive pressure ventilation; PDA, patent ductus arteriosus; CSF, cerebrospinal fluid; LP, lumbar puncture.

Neurological monitoring throughout the hospitalization included serial cranial ultrasound examinations, which revealed no intracranial hemorrhage or other abnormalities. Neurological status was closely assessed for signs of acute bilirubin encephalopathy, with no abnormalities observed in mental status (e.g., lethargy, irritability), muscle tone (e.g., hypertonia, arching), or cry pattern throughout the acute phase. The infant was discharged after a 54-day hospitalization, achieving a discharge weight of 2030 g. Follow-up assessments were conducted at corrected ages of 3, 4, 6, and 12 months. At 4 months of age, neurological examination demonstrated normal muscle tone, age-appropriate primitive reflexes, and no signs of dystonia, athetosis, or gaze palsy. Visual tracking and social smiling were appropriate for age. Brain MRI revealed normal structural integrity without identifiable abnormalities (Figures 3A–I), and concurrent AABR testing showed normal results. At one year corrected age, neurological examination was normal with intact hearing, vision, and motor function. Scores on the Bayley Scales of Infant Development-III were within the normal range (cognitive 95, language 92, motor 90). The timeline of the patient’s clinical course and management is shown in Figure 4.

## Discussion

This report presents an instructive clinical scenario: an extremely preterm infant with septic shock exhibited profound yellow discoloration of CSF. Laboratory analysis revealed an extreme elevation of CSF bilirubin (10 mg/dL), which stood in stark contrast to a concurrently measured serum total bilirubin level that was only minimally elevated (4.4 mg/dL). This profound dissociation illustrates a significant limitation of serum-based monitoring in reflecting severe central nervous system bilirubin exposure in high-risk preterm infants, likely precipitated by sepsis-induced blood–brain barrier disruption. The immediate initiation of intensive phototherapy, prompted by this CSF finding, led to the rapid normalization of CSF bilirubin and a favorable neurodevelopmental outcome. This case suggests that CSF bilirubin could serve as an early biomarker for identifying imminent neurotoxicity risk when serum levels are misleadingly low, thereby highlighting a critical scenario that current management paradigms may not adequately address.

Serum bilirubin remains the cornerstone for guiding phototherapy in neonates, yet its utility as a surrogate for cerebral bilirubin burden is particularly limited in preterm infants due to their inherently immature and dynamic BBB (Hulzebos et al., 2014). In contrast, CSF bilirubin provides a direct window into the CNS compartment, reflecting the concentration of neurotoxic unbound bilirubin. Historical data suggest that CSF bilirubin levels exceeding 0.15 mg/dL may signify encephalopathy risk, even with modest serum elevations (Kulkarni et al., 1989). The invasiveness of lumbar puncture rightly precludes its routine use. This case, however, challenges a passive stance. Here, a diagnostically indicated lumbar puncture serendipitously revealed strikingly yellow CSF, with a bilirubin level orders of magnitude higher than the historical risk threshold, while serum bilirubin was clinically unremarkable. This extreme gradient vividly illustrates how conditions like severe sepsis and acidosis can catastrophically increase BBB permeability (Fan et al., 2022; Su et al., 2025; Barichello et al., 2020; Liu et al., 2025), facilitating substantial bilirubin influx into the CNS independent of serum levels. Therefore, we posit that in high-risk preterm infants—particularly those with conditions known to disrupt BBB integrity—CSF bilirubin measurement could be intentionally integrated into the diagnostic workup when a lumbar puncture is already clinically warranted. This targeted approach could transform CSF analysis from a mere infection screen into a crucial tool for quantifying cerebral bilirubin exposure and refining neuroprotective strategies.

Prolonged PPROM (>20 days) and maternal colonization with ESBL-producing *E. coli* represents important risk factors for ascending intrauterine infection. Although blood cultures were negative, the infant developed early-onset clinical sepsis and septic shock, suggesting a significant systemic inflammatory response. The profound CSF bilirubin elevation observed in our patient, despite low serum levels, can be mechanistically understood through a “double-hit” model targeting the immature BBB. The first hit is the developmental predisposition of preterm infants, characterized by immature expression of tight junction proteins (e.g., claudin-5, occludin), which facilitates bilirubin entry into susceptible brain regions (Ballabh et al., 2005; Saunders et al., 2012; Lochhead et al., 2020; Okumura et al., 2021). The critical second hit in this case was severe sepsis. Inflammatory cytokines associated with sepsis are known to further disrupt BBB integrity, downregulating tight junction proteins and creating a state of dramatically increased permeability (Fan et al., 2022; Su et al., 2025; Barichello et al., 2020; Liu et al., 2025). This synergistic disruption likely allowed massive bilirubin flux into the CNS, decoupling CSF levels from serum concentrations. Consequently, this “double-hit” model underscores that in extremely preterm infants, the triad of extreme prematurity, sepsis (particularly septic shock), and concomitant metabolic acidosis constitutes a clinically recognizable high-risk scenario for a profound dissociation between serum and CSF bilirubin levels, mandating heightened clinical vigilance. Once within the brain, bilirubin exerts neurotoxicity by disrupting mitochondrial function, uncoupling oxidative phosphorylation, and triggering oxidative stress and apoptosis (Hansen et al., 2020; Watchko and Tiribelli, 2013). Concomitant acidosis, as seen in septic shock, can potentiate this neuronal injury (Lai et al., 2020). The successful management of this case appeared to rely on a dual-targeted strategy that not only reduced the bilirubin burden but also facilitated restoration of the blood–brain barrier (BBB). Previous studies have shown that severe sepsis and metabolic acidosis can markedly increase BBB permeability through inflammatory cytokine release, oxidative stress, and endothelial injury (Gao and Hernandes, 2021; Zhao et al., 2015; Barichello et al., 2023). In our patient, the therapeutic interventions directly addressed these pathogenic mechanisms. First, control of infection and inflammation played a central role. Effective antimicrobial therapy combined with hemodynamic support helped eliminate the pathogen and reduce circulating inflammatory mediators, thereby mitigating ongoing inflammatory damage to the BBB. Second, correction of metabolic acidosis may have contributed to the recovery of endothelial integrity. Improved tissue perfusion and oxygenation facilitate normalization of endothelial cell metabolism and restoration of tight junction structure, which are essential for maintaining BBB function. Third, phototherapy may exert an indirect protective effect on the BBB. Although the primary mechanism of phototherapy is the photo-isomerization and enhanced clearance of bilirubin, rapid reduction of circulating unbound bilirubin could decrease its toxic effects on vascular endothelial cells (Palmela et al., 2011). Taken together, the combination of conventional bilirubin-lowering therapy (phototherapy) and aggressive etiological treatment—including infection control and correction of metabolic disturbances—may have synergistically reduced BBB permeability, thereby limiting further entry of neurotoxic substances into the central nervous system.

This case provides a compelling *in vivo* demonstration of the complex kinetics governing bilirubin distribution between serum, CSF, and brain parenchyma. The observed “decoupling” is particularly instructive: at the peak of illness, CSF bilirubin reached 10 mg/dL against a serum level of 4.4 mg/dL; during recovery, CSF levels plummeted while serum bilirubin exhibited a paradoxical rise. This temporal dissociation—where CNS bilirubin burden peaked and resolved out of phase with serum concentrations—suggests regulatory processes beyond passive diffusion across a compromised BBB. We propose that active, compartmentalized mechanisms within the CNS, such as differential cellular uptake/efflux, variable intracerebral binding, or distinct local metabolic clearance pathways, may critically influence neurotoxicity risk (Bockor et al., 2017). This phenomenon underscores that the duration of cerebral bilirubin exposure may be poorly predicted by serum trends alone and highlights a significant gap in our understanding of bilirubin neuropharmacokinetics in sick preterm infants.

The rapid decline of CSF bilirubin underscores the efficacy of our multimodal, pathophysiologically-targeted approach. Intensive phototherapy worked by degrading serum bilirubin and, crucially, by reducing the serum-to-CSF concentration gradient, facilitating bilirubin efflux from the CNS (Hansen et al., 2020). This effect was potentiated by concurrent measures to repair the BBB—aggressive treatment of sepsis and acidosis—and by albumin administration to bind circulating free bilirubin. The resultant decrease in CSF bilirubin from 10 mg/dL to 3.6 mg/dL within four days would be expected to limit the duration of toxic neuronal exposure, a principal factor in the pathogenesis of bilirubin-induced neurologic damage (Watchko and Tiribelli, 2013).

Placing the extreme CSF bilirubin value of 10 mg/dL into context reveals a stark knowledge gap. Existing literature offers limited and dated reference points, largely from term infants, with a historical risk threshold suggested around 0.1–0.15 mg/dL (Kulkarni et al., 1989) and normal values around 0.1 mg/dL (Luz, 1975). It is acknowledged that comorbidities like sepsis lower the threshold for neuronal injury (Hansen et al., 2020; Okumura et al., 2021; Merhar and Gilbert, 2005). Our case dramatically extends the observed spectrum of survivable CSF bilirubin levels in the modern NICU era, provided immediate and comprehensive neuroprotection is instituted. This observation challenges any notion of a universal, absolute toxic threshold and refocuses the question on the dynamic interplay between bilirubin concentration, exposure time, and concurrent brain vulnerability. The current American Academy of Pediatrics (AAP) guidelines, which exclusively rely on serum bilirubin, understandably do not address CSF monitoring due to its invasiveness and the historical lack of robust data (Kemper et al., 2022). However, this case presents a compelling paradox that the guidelines cannot currently address: a neonate with minimal serum hyperbilirubinemia yet at imminent risk of severe bilirubin neurotoxicity, identifiable only through CSF analysis.

This case illustrates a critical clinical dilemma where significant CNS bilirubin exposure occurs with benign serum levels, a scenario not addressed by current serum-based guidelines (Kemper et al., 2022). The profound dissociation we observed, coupled with the established poor correlation between serum and cerebral bilirubin in the context of BBB disruption (Hulzebos et al., 2014), mandates a more nuanced approach. We propose a targeted, evidence-generating strategy: in high-risk preterm infants undergoing lumbar puncture for standard indications (e.g., sepsis rule-out), concomitant CSF bilirubin measurement should be considered to directly assess neurotoxicity risk. This approach is not intended for routine use but to enrich our understanding in complex cases where serum indices are most likely to fail.

It should be noted that pathogen-specific PCR testing was not performed on CSF or nasopharyngeal samples in this case. Although blood and CSF cultures were sterile, the clinical diagnosis of sepsis was supported by significant perinatal infection risk factors and the development of septic shock. The incorporation of molecular diagnostics in future cases may improve pathogen detection in culture-negative infections.

Acknowledging the limitations of this single report and the procedural risks, we underscore the stark evidence gap. Existing CSF bilirubin data are scarce and dated (Kulkarni et al., 1989; Luz, 1975). Therefore, we call for prospective studies to define the predictive value of CSF bilirubin for neurodevelopmental outcomes, establish evidence-based risk thresholds in high-risk populations, and evaluate its potential to refine neuroprotective strategies. Ultimately, integrating such precision biomarkers within comprehensive multidisciplinary care may further improve outcomes for the most vulnerable infants.

## Conclusion

This case demonstrates that in extremely preterm infants, severe central nervous system bilirubin exposure (reflected by elevated CSF bilirubin) can occur with deceptively low serum levels. The favorable outcome following CSF-guided intervention highlights the potential of CSF bilirubin as an early biomarker to identify hidden neurotoxicity risk. Therefore, in selected high-risk preterm infants already undergoing lumbar puncture, measuring CSF bilirubin could provide a direct assessment to guide neuroprotective strategies. This case underscores the need for heightened vigilance regarding bilirubin neurotoxicity in extremely preterm infants with sepsis, even in the absence of significant serum hyperbilirubinemia.

## Data Availability

The original contributions presented in the study are included in the article/supplementary material, further inquiries can be directed to the corresponding author.
